# Dopamine Modulates Persistent Synaptic Activity and Enhances the Signal-to-Noise Ratio in the Prefrontal Cortex

**DOI:** 10.1371/journal.pone.0006507

**Published:** 2009-08-05

**Authors:** Sven Kroener, L. Judson Chandler, Paul E. M. Phillips, Jeremy K. Seamans

**Affiliations:** 1 Department of Neurosciences, Medical University of South Carolina, Charleston, South Carolina, United States of America; 2 Department of Psychiatry and Behavioral Science, University of Washington, Seattle, Washington, United States of America; 3 Department of Psychiatry and Brain Research Centre, University of British Columbia, Vancouver, British Columbia, Canada; Universidade Federal do Rio de Janeiro (UFRJ), Instituto de Biofísica da UFRJ, Brazil

## Abstract

**Background:**

The importance of dopamine (DA) for prefrontal cortical (PFC) cognitive functions is widely recognized, but its mechanisms of action remain controversial. DA is thought to increase signal gain in active networks according to an inverted U dose-response curve, and these effects may depend on both tonic and phasic release of DA from midbrain ventral tegmental area (VTA) neurons.

**Methodology/Principal Findings:**

We used patch-clamp recordings in organotypic co-cultures of the PFC, hippocampus and VTA to study DA modulation of spontaneous network activity in the form of Up-states and signals in the form of synchronous EPSP trains. These cultures possessed a tonic DA level and stimulation of the VTA evoked DA transients within the PFC. The addition of high (≥1 µM) concentrations of exogenous DA to the cultures reduced Up-states and diminished excitatory synaptic inputs (EPSPs) evoked during the Down-state. Increasing endogenous DA via bath application of cocaine also reduced Up-states. Lower concentrations of exogenous DA (0.1 µM) had no effect on the up-state itself, but they selectively increased the efficiency of a train of EPSPs to evoke spikes during the Up-state. When the background DA was eliminated by depleting DA with reserpine and alpha-methyl-p-tyrosine, or by preparing corticolimbic co-cultures without the VTA slice, Up-states could be enhanced by low concentrations (0.1–1 µM) of DA that had no effect in the VTA containing cultures. Finally, in spite of the concentration-dependent effects on Up-states, exogenous DA at all but the lowest concentrations increased intracellular current-pulse evoked firing in all cultures underlining the complexity of DA's effects in an active network.

**Conclusions/Significance:**

Taken together, these data show concentration-dependent effects of DA on global PFC network activity and they demonstrate a mechanism through which optimal levels of DA can modulate signal gain to support cognitive functioning.

## Introduction

Dopamine (DA) modulation of the prefrontal cortex (PFC) plays an important role in cognitive functions, including working memory. Dopamine modulation of working memory performance and the associated task-related neuronal activity within the PFC follows an inverted U-shape dose-response curve, with optimal signal processing at the peak of the inverted-U function; [1]–[5]. Thus, DA can have both facilitatory and suppressive effects on cortical neurons, and it has been suggested that the concentration-dependent effects of DA *in vivo* depend upon both the prevailing “tonic” DA concentration as well as fluctuations in DA concentrations from “phasic” release [6], [7].

Mechanistic studies in-vitro have identified a multitude of pre- and postsynaptic as well as intrinsic ionic currents through which DA modulates neural activity (reviewed in [8]). Virtually all of these currents can produce non-linear changes in membrane potential that involve multiplicative and/or opposing actions, and are expected to have vastly different effects depending on whether a neuron is at rest or is embedded in an active network. In addition, DA has receptor- and concentration-specific effects that are consistent with the inverted-U concept to explain its actions [9], [10]. However, acute slice preparations are largely devoid of the ongoing network activity and functionally significant DA tone, which can influence neuronal responses [11]–[14]. Thus, how the effects of DA on intrinsic membrane excitability and synaptic connections between various cell-types interact in an active recurrent network is difficult to predict from observations of each of these components in isolation.

Here, we used patch-clamp recordings in organotypic slice co-cultures of the PFC, the hippocampus (Hipp), and the midbrain containing the ventral tegmental area (VTA) to investigate the effects of varying levels of DA on recurrent synaptic activity in the PFC in the presence or absence of a tonic DA level. These cultures possess both an intrinsic source of DA from the VTA, as well as intrinsic network activity in the form of “Up-” and “Down-states” [15], [16]. We tracked the membrane potential, evoked spiking behavior, and the response to synaptic inputs in PFC pyramidal neurons under conditions designed to alter both tonic and phasic levels of DA. Our results show that DA can independently alter spontaneous network activity (the Up-state) and a superimposed synaptic “signal”, consistent with the idea that DA modulates the signal-to-noise ratio (S∶N) in active networks. The effects on both Up-states and evoked synaptic potentials were concentration-dependent, with activity declining at higher concentrations. Furthermore, the concentration-dependent effects of DA on Up-states were influenced by the presence or absence of tonic DA levels in the cultures. Taken together, these data confirm important aspects of the hypothesized inverted-U DA dose-response curve and provide further support for the idea that DA optimizes signal processing in active cortical networks by improving the S∶N ratio.

## Methods

All animals were handled in strict accordance with the Guidelines for the Care and Use of Animals published by the USPHS and followed procedures approved by MUSC's Institutional Animal Care and Use Committee (protocol number AR2605).

### Preparation of triple slice co-cultures

**Figure 1:**
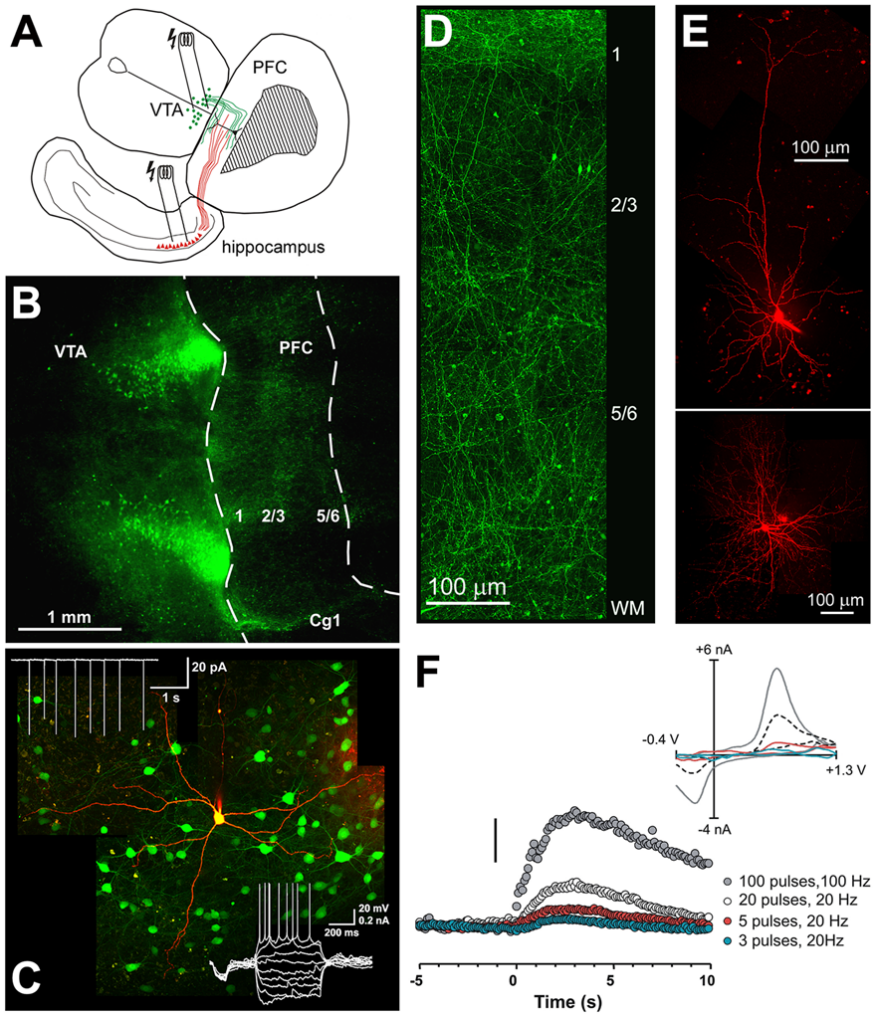
Properties of the organotypic triple co-culture system and the DA innervation of the PFC as demonstrated by tyrosine-hydroxylase (TH) containing fibers. A) Schematic representation of the triple co-culture consisting of the PFC, VTA, and hippocampus. Electrical stimulation of the afferents from the VTA (indicated as green lines) or ventral hippocampus (red lines) induces Up-states in the PFC. B-D) Photomicrographs illustrating putative DAergic (TH-positive) neurons in the VTA and the distribution of TH-fibers in the PFC. Co-cultures were made from mice expressing green fluorescent protein under the control of the TH gene promoter. C) Properties of putative DAergic (green TH-positive) neurons in the VTA. Cell-attached recordings (top left inset) show that DA neurons are tonically active. Bottom right inset: Membrane properties and firing response in whole-cell mode in response to a series of hyperpolarizing and depolarizing current steps (−150 to+120 pA). The recorded cell was filled with Alexa 594 after break-in. D) shows the laminar distribution of fibers in the PFC. E) Morphological properties of a pyramidal cell (top) and interneuron in the PFC of organotypic co-cultures. Cells were loaded with Alexa 594 during recording and visualized using series of confocal images. Images are montages of convoluted z-stacked images at 40× magnification in C-F. All images were contrast-enhanced for clarity. F) Electrochemical detection of phasic DA release in the PFC following stimulation of the VTA. Stimulation trains (3–100 pulses) were initiated at time 0, and evoked an increase in extracellular DA. Scale bar is 200 nM. The insert shows background-subtracted cyclical voltammograms taken at the peak of the response for each of the stimulations. Abbreviations: VTA, ventral tegmental area; PFC, prefrontal cortex, Cg1, cingulate cortex; WM, white matter.

Co-cultures were made from mice at postnatal days 2–4. We used both C57BL/6 and mice expressing green fluorescent protein (GFP) under the control of the tyrosine hydroxylase (TH) gene promoter (c.f. Fig. 1; animals for establishing the TH-GFP breeding colony were kindly provided by Dr Hideyuki Okano, Keio, University; [17]). Pups were anesthetized by hypothermia and decapitated. Sections (325 µm thick) containing the prelimbic and infralimbic regions of the PFC, the level of the midbrain containing the VTA, and the ventral hippocampus were prepared on a vibratome (Leica VT 1000, Nussloch, Germany) in ice-cold sucrose-substituted solution (in mM): 200 sucrose, 1.9 KCl, 6 MgCl2, 0.5 CaCl2, 10 glucose, 0.4 ascorbic acid, 10 HEPES. Slices were placed close to each other on a Millipore millicell insert in a six-well culture dish. The plating media consisted of: 50% basal medium Eagle, 25% Earle's balanced salt solution, 25% horse serum plus 6.5 mg/ml glucose, 25 mM HEPES–NaOH (pH 7.2), 100 µg/ml streptomycin and Glutamax for the first 3 days. Every 3–4 days thereafter, inserts were placed in a fresh dish with 850 µl of the same media as above, except 70% basal medium Eagle, 25% Earle's solution and 5% horse serum were substituted. After 15 days, 10 µl of 5-fluoro-2-deoxyuridine (0.08 mM) plus uridine (0.2 mM) in MEM was added to the media to prevent cell division and glial overgrowth.

### Electrophysiological procedures and data analysis

After a minimum of 16 days in culture, individual co-cultures were transferred to a recording chamber where they were bathed in artificial cerebrospinal fluid (ACSF) consisting of (in mM): 125 NaCl, 3.8 KCl, 25 NaHCO_3_, 1.2 CaCl_2_,1 MgCl_2_, 10 dextrose and 0.4 ascorbic acid, saturated with 95% O2–5% CO_2_ at 37°C. Whole-cell recordings were obtained with an Axon Multiclamp-200 amplifier from neurons in deep cortical layers identified using infrared differential-interference contrast optics and videomicroscopy on a Zeiss FS-2 microscope. For current-clamp recordings, electrodes (3–5 MΩ open tip resistance) were filled with a solution containing (in mM): 120 K-gluconate, 10 HEPES, 10 KCl, 10 NaCl, 4 ATP-Mg, 0.3 GTP-Na, 14 phosphocreatine and 0.04 Alexa 594, pH 7.2 (KOH). Signals were low-pass filtered at 3 kHz, and digitized at 5 kHz during voltage-clamp- and current-clamp recordings. Data were stored on PC for off-line analysis using HEKA Tida software, custom LabView software, or Axograph X for Windows (Axograph, Sydney, AUS). The morphology of pyramidal and non-pyramidal cells, respectively, was confirmed using high-resolution confocal imaging of Alexa Fluor 594.

Intrinsic membrane properties and the evoked firing pattern were used to distinguish potential subtypes of deep-layer PFC neurons. Therefore, series of hyperpolarizing and depolarizing current steps (500 ms duration; 10–20 pA increments at 0.3 Hz) were delivered from resting membrane potential to evoke spike firing at various steady-state membrane potentials. Evoked firing by somatic current injection served as an internal control to determine changes in neuronal excitability following DA application, and it aided comparison with previous studies in acute brain slices. Comparisons of changes in the number of evoked spikes were made at a current level that reliably produced repetitive firing under control conditions.

Up-states were evoked synaptically by electrical stimulation of the VTA, the ventral hippocampus, or the contralateral PFC, as indicated, using bipolar concentric tungsten electrodes (TM33CCNON, World Precision Instruments). Current pulses (2–9, 0.12 ms duration each, at 20 Hz) were generated by stimulus isolation units (A360, World Precision Instruments), triggered digitally by our acquisition software.

In order to assess changes in Up-states, we measured the total duration of the Up-state (between the start of the synaptic stimulation and the point when the membrane potential returned to baseline values) and the number of spikes during the first 500 ms of the Up-state. Under our baseline conditions, all Up-states were longer than 500 ms. Therefore, restricting the spike count to the first 500 ms served as a way to minimize the confounding influence of changes in Up-state duration on the spike count.

In experiments in which we studied DA modulation of synaptic short-term plasticity both during the Up-state and Down-state we placed a theta-glass electrode in the deep layers within 100 µm lateral to the recorded cell. Theta-glass electrodes were filled with ACSF and connected to a stimulus isolation unit via silver wires to evoke small excitatory postsynaptic potentials (EPSPs). The glutamatergic nature of the evoked postsynaptic potentials was confirmed at the end of the experiments by bath application of the AMPA receptor blocker 6-cyano-7-nitroquinoxaline-2,3-dione (CNQX, 20 µM) (see Results). Trains of 15 pulses at 20 Hz were delivered every 30–45 s from the resting membrane potential. We measured both the amplitude of each individual EPSP and the area under each EPSP in the train relative to the initial voltage before the train onset. Measuring the area under each EPSP accounts for the amount of residual depolarization due to the summation of EPSPs.

After collection of baseline data, DA was bath-applied for 2–3 minutes. Each culture was exposed to only a single application of DA. When the effects of DA antagonists were examined, the D1 antagonist R-[+]-7-chloro-8-hydroxy-3-methyl-1-phenyl-2,3,4,5-tetrahydro-1H-3-benzazepine (SCH23390), or the D2 antagonist (±)-Sulpiride (both from Sigma, St. Louis, MO) were bath-applied at least 10 minutes prior to application of DA and continued to be present throughout the remainder of the experiment. Some experiments were conducted in the presence of 20 µM CNQX, 10 µM of the NMDA receptor antagonist (±)-3-(2-Carboxypiperazin-4-yl)propyl-1-phosphonic acid (CPP), or the GABA_A_ receptor antagonist picrotoxin (75 µM), as indicated.

For statistical comparisons, electrophysiological parameters were measured at multiple time points before and after drug application and averaged for each experimental condition (a minimum of 5 repetitions over 10 minutes for each condition). Comparisons were performed using analysis of variance (ANOVA) and two-tailed, paired t-tests as indicated (differences of alpha ≤0.05 were considered significant). For multiple *post-hoc* comparisons the alpha-level was Bonferroni-adjusted as indicated. All data are presented as means±SEM. All statistical comparisons were performed on the raw data, but in several figures we depict results as percent changes over baseline values to aid comparison across multiple treatment groups.

### Measurement of DA concentration in the culture media

For analysis of DA level in the culture media, an aliquot of the media (150 µl) was pre-cleared by centrifugation at 15,000 g for 10 min and the supernatant passed through a 3,000 KDa size exclusion spin-column. For measurement of DA by HPLC using electrochemical detection, twenty µl of each recovered sample was injected onto a SPER C18 reverse-phase narrowbore column (100×2.1 mm, Princeton Chromatography, Cranbury, NJ) using an Alcott Model 718 AL Autosampler (Norcross, GA). Flow rate through the column was 0.23 ml/min and controlled by a Model LC1120 isocratic pump (GBC Scientific, Hampshire, IL). A Decade Amperometric Electrochemical Detector (Antec Leyden, The Netherlands) was set to a working potential of+400 mV. Mobile phase consisted of 6% methanol, 65 mg/l octane sulfonic acid, 40 mg/l EDTA, 0.05 M phosphoric acid, 0.05 M citric acid; pH = 3.0. Data were quantified by comparing peak areas against those of a four-point calibration of DA standards (0, 1, 5 and 10 pg/µl).

### Electrochemical detection of dopamine in the slice co-culture

Changes in extracellular DA concentration within the PFC of the slice co-culture were measured using fast-scan cyclic voltammetry (FSCV) with carbon-fiber microelectrodes (7 µm diameter; ∼25 µm exposed surface; Goodfellow, PA). The potential at the microelectrode was held at −0.4 V vs. a Ag/AgCl reference electrode and then linearly ramped to+1.3 V and back (400 V/s) every 100 ms. For analyte identification, oxidation currents during a voltammetric scan were plotted against the applied potential to yield a cyclic voltammogram. For quantification of changes in dopamine concentration over time, the current at its peak oxidation potential was plotted for consecutive voltammetric scans. Waveform generation, data acquisition and analysis were carried out on a PC-based system using software written in LabVIEW (National Instruments, TX) that controlled a custom built voltammetric amplifier.

## Results

### Cortical Dopamine innervation in VTA-PFC-Hipp co-cultures

Coronal slices of the frontal cortex and the caudo-ventral Hipp were co-cultured with a midbrain slice containing the VTA to explore the impact of DA innervation on cortical physiology. To verify a strong DA innervation of the PFC, a subset of co-cultures were made from mice expressing GFP under the control of the TH gene promoter and the GFP signal was visualized using confocal fluorescent microscopy (Fig. 1). Similarly, in separate cultures prepared from wildtype mice, we used immunohistochemistry for TH to identify DAergic neurons and fibers (data not shown). In all cases, numerous TH+neurons were observed in the VTA that extensively innervated the co-cultured PFC slice (Fig. 1C-E), replicating our own previous findings [16], [18]. Also consistent with our previous observations [18], the TH-GFP+cells in the VTA of these slice co-cultures were spontaneously active in cell-attached recordings (Fig. 1C, top left inset) thereby providing a DAergic tone to PFC neurons.

We analyzed the incubation media from the culture wells using HPLC with electrochemical detection in order to provide an indication of the DA levels at equilibrium after 15+days in culture. The DA levels in the culture media were 8.6±3.4 nM (n = 12). Although this measurement is not likely a true estimate of the tissue content of DA, it nevertheless indicates that DA was present at levels close to those measured in-vivo using microdialysis when corrected for probe recovery and depletion around the probe [19], [20]. We also tested whether the DA fibers in the PFC were able to release DA in response to electrical stimulation of the VTA. Figure 1F shows data that stimulation of the VTA could elicit measurable DA release events as detected by fast-scan cyclic voltammetry using a carbon electrode in the PFC. Dopamine was detectable by this means in 6 of 10 cultures tested, with a detection limit of ∼40 nM. Peak extracellular DA concentration following stimulation ranged from 50 nM for 1 pulse to 570 nM for 100 pulses (100 Hz). These data demonstrate that DA release in the co-cultures occurs via both tonic and phasic processes and thus closely mirror the in-vivo conditions.

### Up-states in co-cultures require activation of AMPA and NMDA receptors

**Figure 2:**
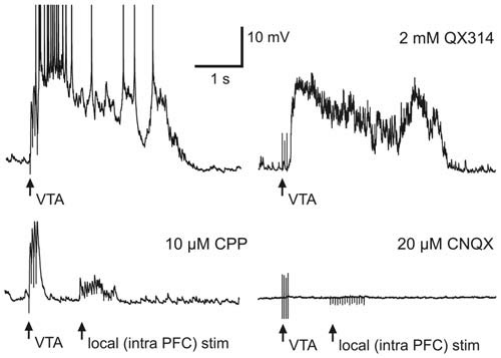
Cortical Up-states in organotypic co-cultures are a network phenomenon. The membrane potential of cortical neurons in PFC-Hipp-VTA co-cultures alternates between a hyperpolarized Down-state close to the resting membrane potential and a depolarized Up-state during which action potential firing occurs. Up-states could be evoked synaptically by short burst stimulation of the VTA, the hippocampus, or the contralateral PFC, respectively (see text for details). Inclusion of the Na^+^ channel blocker QX-314 in the recording pipette did not alter the occurrence or duration of Up-states. In contrast, glutamatergic transmission at both non-NMDA and NMDA receptors is required to initiate and sustain Up-states, respectively. In the presence of the NMDA receptor antagonist CPP (10 µM; n = 7), stimulation of the VTA or the hippocampus evoked large EPSPs, but these failed to evoke recurrent activity and Up-states. Bath application of CNQX (20 µM; n = 18) blocked all evoked responses following stimulation of either the VTA or the hippocampus, as well as a large portion of locally evoked PSPs.

Electrical stimulation of the VTA evoked Up-states in the PFC, which we recorded in current clamp mode from deep layer neurons (Fig. 2). As shown above the brief burst stimulation used to initiate these Up-states also evoked measurable DA transients in the PFC; however, both in-vivo [11], [21] and in-vitro [16], [22] recurrent activity during Up-states primarily depends on the balance of excitation and inhibition, and several lines of evidence suggest that NMDA receptors play a crucial role in the maintenance of the Up-state. Accordingly, bath application of either the non-NMDA receptor antagonist CNQX (20 µM; n = 18), or the NMDA antagonist CPP (10 µM; n = 7) completely blocked all evoked Up-states (Fig. 2). In the presence of CPP, post-synaptic potentials (PSPs) could still be evoked by stimulation of long-range afferents from the VTA (Fig. 2, bottom left panel) or the hippocampus (not shown), as well as by local stimulation within the PFC. Bath application of CNQX blocked all evoked responses following stimulation of either the VTA or the hippocampus, as well as the majority of locally evoked PSPs (Fig. 2, bottom right panel). Finally, blockade of sodium spikes in the recorded neuron by addition of 2 mM QX314 to the intracellular recording solution did not affect the generation and maintenance of Up-states (Fig. 2, top right panel). Taken together, these results show that cortical Up-states in slice co-cultures represent a network phenomenon that requires activation mediated by non-NMDA receptors and which is sustained by a significant contribution of NMDA receptors. The remainder of the study focused on how DA can modulate these largely glutamate mediated up-states.

### Effects of varying concentrations of exogenous Dopamine on cortical Up-states

In the first set of experiments investigating DA modulation of activity states, we examined the effects of increasing ambient DA above the intrinsic background levels by bath application of known concentrations of DA. We chose to focus on the effects resulting from application of DA itself because DA is the endogenous agonist and because of the complex cooperative and non-cooperative interactions among DA receptor subtypes that may also vary with time and concentration [25]–[27]. We examined a range of concentrations (10 nM – 50 µM) that have previously been utilized in acute slice preparations to study the effects of DA on intrinsic membrane excitability [28]–[31] and synaptic transmission [10], [32], [33] in the PFC.

**Figure 3:**
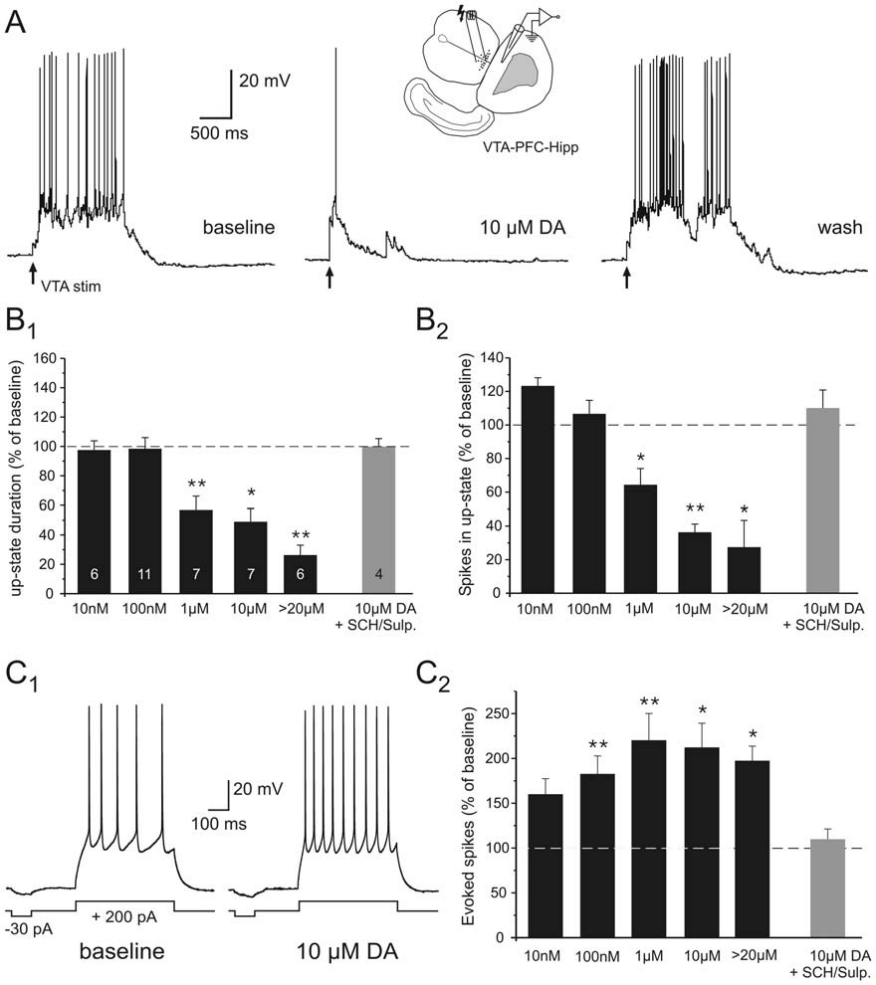
Dopamine-modulation of cortical Up-states is concentration-dependent. DA was bath-applied to VTA-PFC-Hipp co-cultures and Up-states were evoked by VTA stimulation (see insert). A) Representative traces showing the effects of high (10 µM) DA on VTA-evoked Up-states. B) At concentrations of 1 µM exogenous DA or higher the duration and number of spikes during the initial 500 ms of the Up-state were significantly reduced. These effects on Up-states were abolished when DA receptors were blocked by combined pre-application of the D1 receptor antagonists SCH 23390 and sulpiride (5 µM each) to the bath for 10 minutes before application of DA (10 µM). C) In marked contrast to the reductions in Up-state duration and action potential firing due to network activity, the number of spikes evoked by somatic current injection was consistently increased across a wide range of exogenous DA concentrations, starting at 100 nM. C1) In the presence of DA, the same cell as shown in A) displays a significant increase in evoked spikes in response to a square pulse current injection. C2) Summary graph of the effects of various bath-applied DA concentrations on spike firing evoked by somatic current injection. Similar to the effects on Up-states shown in B), increases in current-evoked spike firing depended on DA receptor activation, and accordingly pre-application of SCH-23390 and sulpiride blocked the effects of 10 µM DA. Statistical comparisons used paired t-tests after repeated measures ANOVA. Levels of significance for multiple comparison were * P<0.01, and ** P<0.005. The number of cells in each group used for comparisons in B) and C) are indicated in B1. The same cells were used for measurements in B) and C).

Repeated measures ANOVA revealed a significant interaction between the exogenous DA concentration and the changes in each group for both the duration of Up-states (F = 10.46; P<0.0001; df = 31) and the number of spikes during the first 500 ms of the Up-state (F = 7.4; P<0.0001; df = 31) in PFC-Hipp-VTA co-cultures. *Post-hoc* analysis using paired t-tests showed that both measures were significantly decreased at concentrations equal to or higher than 1 µM DA in the bath (Fig. 3A, B; Bonferroni-corrected level of significance for multiple comparisons was P<0.01). The majority of cells showed at least a partial wash-out effect and return towards baseline values ∼20 minutes after DA was washed out of the bath.

In contrast to the effects on Up-state duration and the number of spikes in the Up-state, the number of spikes evoked by somatic current injection from the Down-state was reliably increased following bath application of all but the lowest concentration (10 nM) of DA (Fig. 3C). Furthermore, consistent with previous studies in acute slices [30], [31], the effects of DA on evoked spike firing were long lasting and often outlasted the duration of the recording (not shown). Both the effects on Up-states and on current-evoked firing were specific for DA receptor activation, as they were abolished when DA receptors were blocked (Fig. 3B, C). Although these agents had no effects under basal conditions (see below), combined pre-application of the specific D1 receptor antagonist SCH-23390 (5 µM) and the D2 receptor antagonist sulpiride (5 µM) for 10 min prevented the effects of exogenous DA on Up-state duration and spike count (n = 4). Similarly, the number of spikes evoked by somatic current injection did not change when DA was applied in the presence of DA receptor antagonists. Taken together, these results indicate that by themselves changes in intrinsic excitability do not accurately predict how DA influences synaptic activity and spiking behavior in an active network. Furthermore, our finding that high concentrations of exogenous DA (i.e., ≥1 µM) depressed activity in the network despite robust increases in intrinsic membrane excitability, suggests that elevations of DA beyond the normal levels in the co-culture (as a result of the combination of tonic and phasic release of DA from midbrain neurons) can alter the balance of excitation and inhibition that characterizes cortical Up-states under control conditions.

**Figure 4:**
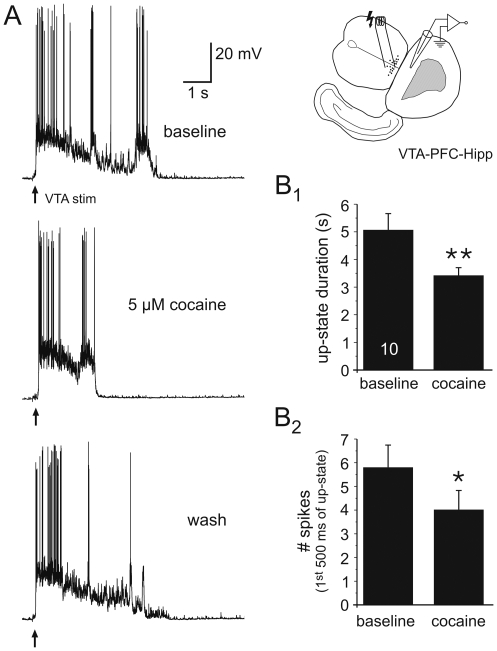
Cocaine enhances endogenous DA activity to reduce VTA-evoked Up-states. A) Representative traces illustrating the effects of 5 µM cocaine on cortical Up-states evoked by VTA-stimulation. B) Altering DA transmission with cocaine (5 or 10 µM, N = 10) resulted in transient reductions in Up-state duration and spike number during the Up-state (Level of significance * P<0.05, and ** P<0.01, compared to baseline, paired Student's t-tests).

To demonstrate that endogenous DA in the cultures is also able to produce a similar effect as bath applied DA, we applied cocaine (5 or 10 µM, n = 10) to block catecholamine reuptake and thereby enhance the endogenous levels of extracellular DA. In the presence of cocaine, the duration and spike count of VTA-induced cortical Up-states were also significantly reduced (Fig. 4). This further demonstrates that increasing DA levels above a previously established level in the co-culture can alter Up-state properties.

### Reducing dopaminergic tone in the co-cultures alters the network response to bath application of dopamine

**Figure 5:**
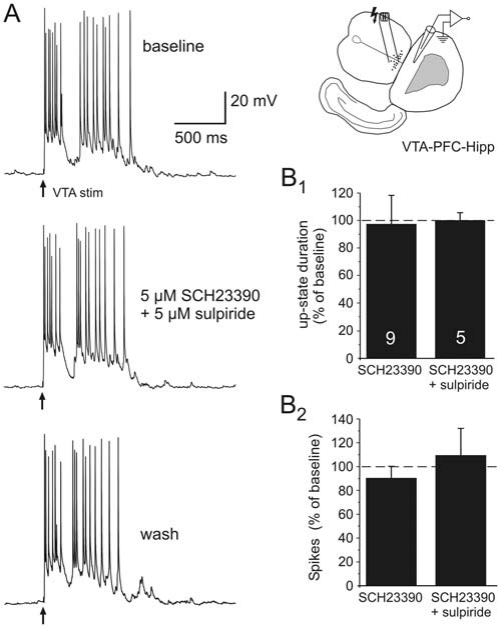
Acute blockade of DA receptors does not affect properties of cortical Up-states in VTA-PFC-Hipp co-cultures. A) Representative traces of cortical Up-states synaptically evoked by brief burst stimulation of the VTA (2–6 pulses at 20 Hz), before (top), during (middle) and 20 minutes after bath application of the DA receptor antagonists SCH23390 and sulpiride (both 5 µM). The insert shows a diagram of the recording configuration with the stimulation electrode in the VTA and the recording electrode in the PFC. B) Bath application of either the DA D1 receptor antagonist SCH23390 (5 µM) alone (n = 9), or in combination with the D2 receptor antagonist sulpiride (5 µM; n = 5) had no significant effect on Up-state duration, or the number of spikes during the first 500 ms of VTA-evoked Up-states.

**Figure 6:**
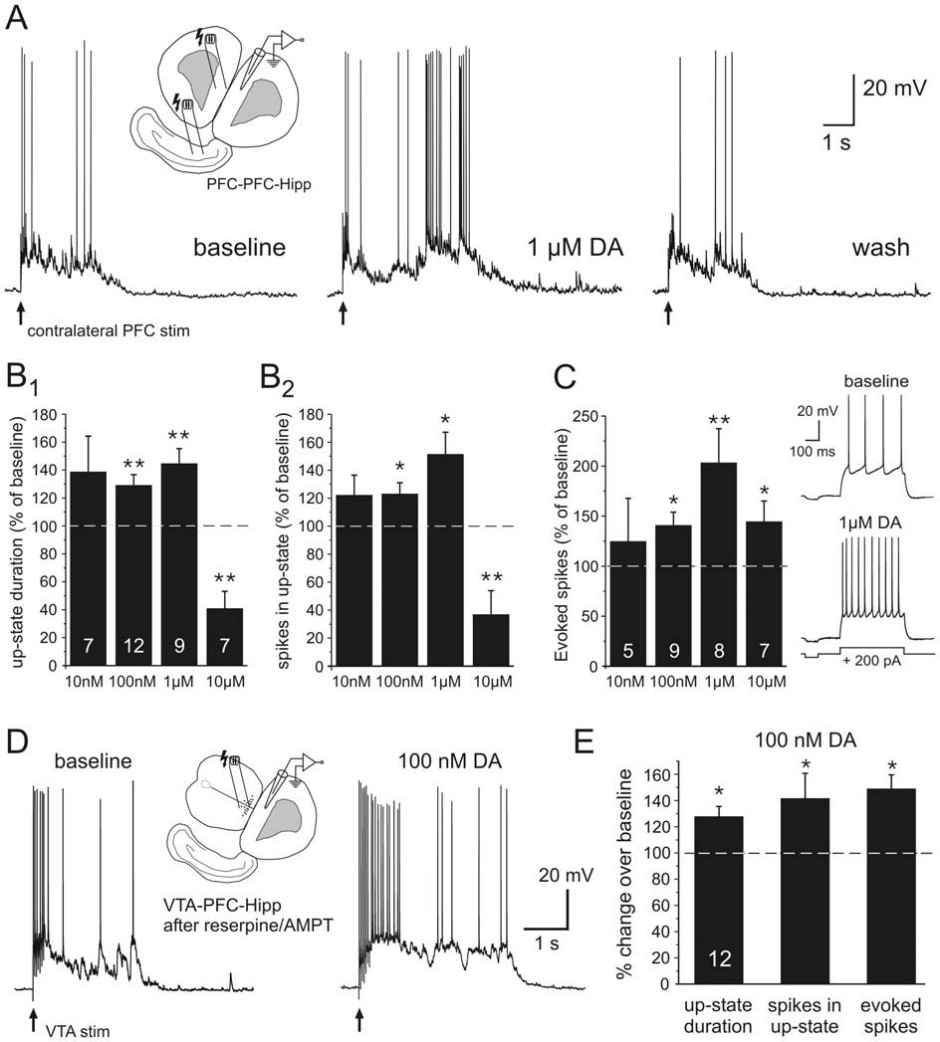
In co-cultures that show no or reduced DAergic tone bath application of DA can increase Up-state duration. A-C) PFC-PFC-Hipp co-cultures were prepared to study the acute effects of DA in the absence of DAergic innervation from the midbrain. Up-states were evoked by electrical stimulation of either the ventral Hipp (n = 23) or the contralateral PFC (n = 12) and the data were pooled (see text for details). The insert in A) shows a diagram of the 2 possible recording configurations. A) Representative traces of Up-states evoked by stimulation of the contralateral PFC before, during and after bath application of 1 µM DA. B) Summary graph of the effects of various doses of bath-applied DA on Up-states in PFC-PFC-Hipp co-cultures. At low to moderate doses (100 nM – 1 µM) DA augmented Up-state duration (B1) and the number of spikes in evoked Up-states (B2). Further increasing exogenous DA concentrations (10 µM) significantly shortened Up-states and the number of spikes in the Up-state, similar to the effects observed in VTA-PFC-Hipp co-cultures. C) Summary graph of the effects of various bath-applied DA concentrations on spike firing evoked by somatic current injection. With the exception of the lowest dose (10 nM) DA consistently increased the number of spikes evoked by somatic current pulses. Statistical comparisons used paired t-tests after repeated measures ANOVA. Levels of significance for multiple comparison were * *P*<0.0125, and ** *P*<0.00625. The numbers of cells in each group used for comparisons are indicated in B1 and C), respectively. D) DA levels in VTA-PFC-Hipp cultures were reduced by adding reserpine (10 µM) and AMPT (100 µM) to the culture media for 5 hours prior to recordings. Up-states were evoked by VTA stimulation and 100 nM DA were bath applied. E) In co-cultures in which DA release was reduced over several hours application of 100 nM DA significantly increased the duration of Up-states as well as the number of spikes in the Up-state. The number of spikes evoked by somatic current injection was also increased (paired t-tests; n = 12).

Next we employed various strategies to test the effects of reducing or removing the background DA tone on Up-states. The first series of experiments tested the effects of acute blockade of DA receptors by bath application of either the D1 antagonist SCH23390 (5 µM) alone (n = 9), or a combination of SCH23390 and the D2 receptor antagonist sulpiride (5 µM; n = 5). None of these manipulations affected Up-state duration or the number of spikes during the first 500 ms of Up-states evoked by VTA stimulation (Fig. 5). This suggested that while adding DA on top of the background levels could significantly impact Up-state properties (Figs. 3, 4), the initiation and maintenance of cortical Up-states does not seem to be acutely modulated by the tonic level of DA found in the cultures, a situation that mirrors findings in the striatum in-vivo [23], [24] and our own previous findings in-vitro [18; but see 34 for striatum]. However, the background levels of DA may still influence the network response to changing levels of exogenous DA. In order to address this issue we examined the effects of exogenous DA on cultures that lacked the VTA-containing midbrain slice and therefore background DA levels. Because Up-states are a network phenomenon and require a critical mass of synaptic connections [35], we included a second prefrontal cortical section in place of the VTA section to ensure that the PFC slice from which recordings were obtained received comparable degrees of afferent innervation across all groups. In these corticolimbic cultures (i.e., PFC-PFC-Hipp), Up-states could be evoked reliably by electrical stimulation of either the contralateral PFC (n = 12) or the ventral hippocampus (n = 23). Both groups showed qualitatively similar responses to the application of DA (see below) and were therefore pooled for further analysis. Under baseline conditions, no significant differences were observed in the number of spikes and the duration of evoked Up-states among neurons recorded from co-cultures that lacked the VTA (spikes: 9.42+/−1.28; duration: 2806.7+/−293.1 ms; N = 35) and those that contained the VTA (spikes: 8.56+/−1.24; duration: 3476.3+/−307.5; N = 75) again showing that the tonic background level of DA on its own had little impact on Up-states. However when various concentrations of DA were added to PFC-PFC-HIPP co-cultures, notable differences from VTA containing cultures were observed. Repeated measures ANOVA revealed a significant interaction between the exogenous DA concentration and the changes in each group for both the duration of evoked Up-states (F = 20.09; P<0.0001; df = 31) and the number of spikes during the first 500 ms of the Up-state (F = 9.72; P<0.0001; df = 31). *Post-hoc* analysis showed that Up-states were significantly prolonged and the number of spikes increased at extracellular concentrations of 100 nM and 1 µM DA (Fig. 6A, B). Higher concentrations of DA (10 µM) again dramatically reduced the duration and number of spikes in evoked Up-states (Fig. 6B). Therefore unlike in VTA containing cultures, in the cultures lacking the VTA low concentrations of exogenous DA were able to increase Up-states. This suggests that one function of the background DA tone may be to dampen overall excitability in the network by preventing elevations in DA levels from non-selectively increasing in Up-states.

A potential confound of these results using PFC-PFC-HIPP co-cultures is that the circuitry underlying Up-states may differ from that in VTA-PFC-Hipp cultures. The lack of DA innervation for an extended period of time during development may further contribute to these potential differences. In order to address these potential confounds, we allowed VTA-PFC-HIPP cultures to develop as usual for 16–25 days, but prior to the electrophysiological experiments pretreated them with a cocktail of reserpine and alpha-methyl-p-tyrosine (AMPT). Reserpine disrupts vesicular storage of DA and thus leads to depletion of DA from the terminal, while AMPT limits the amount of newly synthesized DA in the cytosol via direct inhibition of tyrosine hydroxylase [36]. Reserpine (10 µM) and AMPT (100 µM) were dissolved in DMSO (0.5% final concentration in the media) and added to the culture media for a minimum of 5 hours prior to recordings. The low levels of DMSO in the media had no apparent effect on the membrane properties of the recorded neurons or the ability to evoke up-states via VTA stimulation (Fig. 6D). The properties of Up-states evoked by VTA stimulation in DA-depleted cultures were comparable to those evoked in untreated cultures (duration: 4382.3±794 ms; spikes 4.81±1.25; n = 12). Bath application of 100 nM DA to reserpine/AMPT pretreated cultures had qualitatively similar effects to those seen in PFC-PFC-HIPP cultures in that it increased up-state duration and the number of spikes in the up-state (Fig. 6E). Therefore, since the effects of reserpine/AMPT were similar to the effects observed in VTA lacking cultures, it indicated that the increase in Up-states was not an artifact of the preparation but an effect that emerges when low concentrations of DA are applied in the absence of a background tone. Finally, as in previous experiments, the number of spikes evoked by somatic current injection was similarly increased by bath application of DA (Fig. 6E).

Taken together, results from the VTA-PFC-Hipp and PFC-PFC-Hipp co-cultures demonstrate that DA can modulate recurrent network activity in the PFC, and suggests that the direction of this modulation depends at least partially on the presence of a background DAergic tone. In VTA-PFC-Hipp cultures, spontaneous activity of VTA neurons supplied DAergic tone while the short burst stimulation of the VTA used to elicit Up-states in the PFC provided phasic DA release. Under these conditions, bath application of DA at a concentration of 1 µM or higher lead to robust reductions in Up-state duration and spike firing. In contrast, in co-cultures that lacked intrinsic DA tonic and phasic DA release from the VTA, bath application of DA at a concentration of 1 µM or lower had the opposite effect as it increased Up-state duration and spike firing.

### Dopamine modulation of synaptic short-term dynamics and EPSP-spike coupling

**Figure 7:**
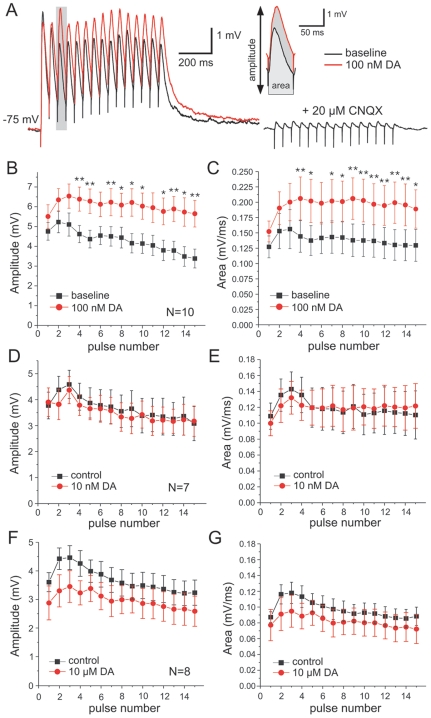
Dopamine modulation of synaptic short-term plasticity in the Down-state. A) Representative traces of EPSPs under baseline conditions (black trace) and following bath application of 100 nM DA. Trains of EPSPs (15 pulses at 20 Hz) were evoked by local stimulation of afferents in the PFC in PFC-Hipp-VTA co-cultures. Under control conditions trains of EPSPs typically showed a mixture of synaptic depression and summation. The glutamatergic nature of the synaptic response was confirmed at the end of the experiment through bath application of the AMPA antagonist CNQX (20 µM; right trace) Traces represent averages of 20 sweeps. The insert illustrates the measurements (amplitude and area) obtained for each EPSP in the train. The EPSPs shown in this example are indicated by the shaded area in the train on the left. B, C) Dopamine (red symbols) at 100 nM increased both the amplitude B), and area under the EPSP C) over baseline values (black symbols). The DA-induced changes in EPSP amplitude and area became significant after short repetitive stimulation, starting with the 4^th^ pulse. Statistical comparisons used paired t-tests after repeated measures ANOVA (* *P*<0.0033, and ** *P*<0.00165). D, E) The low concentration of 10 nM DA did not alter EPSP amplitude D) or area under the curve E). At high levels of exogenous DA (10 µM) the amplitude of the EPSPs, F), was reduced across all pulses in the train (repeated measures ANOVA; *P*<0.05). G) The area under the curve showed a similar trend but this change did not reach significance in our sample (n = 8).

Catecholamines, and particularly DA, are believed to increase the efficiency of cortical processing by augmenting the signal-to-noise ratio or gain within cortical networks [4], [37]–[39]. Specifically, it has been hypothesized that by increasing the effects of strong, sustained depolarizing inputs relative to background firing [40], [41], DA augments task-related activity. One way that DA could achieve this is to alter the response to trains of inputs so that they would produce a more prolonged depolarization that in turn would aid the persistent firing associated with working memory. Previous experimental studies in acute slice preparations [32], [42] have described effects of exogenous DA on short-term synaptic plasticity that are consistent with this idea. Here, we examined whether similar effects are observed in co-cultures in the presence of a functional DA tone and how these changes could influence the effectiveness of EPSPs to induce spike firing during the Up-state. To this end, we again used PFC-HIPP-VTA co-cultures and evoked trains of PSPs (15 pulses at 20 Hz) by local stimulation of afferents within 100 µm lateral of the soma of the recorded cell. Synaptic responses in the train typically showed a mixture of synaptic depression and summation (Fig. 7A). Under our recording conditions, both inhibitory GABAergic and excitatory glutamatergic synaptic responses produced depolarizing postsynaptic potentials from the Down-state. In order to verify the nature of the synaptic connection at the end of the experiment, we applied 20 µM CNQX to the bath. This invariably eliminated the Up-state and the synaptic response from the hippocampus (c.f. Fig. 2). In the majority of cells tested (25 out of 32), this also completely blocked the locally evoked synaptic inputs (Fig. 2, bottom right). The synaptic potentials in the remaining 7 cells contained a significant GABAergic component (∼40–100% of the PSP under baseline conditions) that was blocked by subsequent bath application of the GABA_A_ receptor antagonist picrotoxin (75 µM), and these cells were not considered for further analysis.

After recording a minimum of 20 EPSP trains in the Down-state, we evoked Up-states in the PFC via brief burst stimulation of the Hipp (3–5 pulses at 20 Hz) and repeated the local synaptic stimulation in the presence of these Up-states. The trains of EPSPs were timed such that they occurred at least 500 ms (but typically more than 1 s) after the onset of the Up-state. We obtained at least 10 pairings of EPSP trains with hippocampus-evoked Up-states before we recorded another 5–10 EPSP trains during the Down-state alone. This was done to ensure that pairing the EPSPs with the Up-state by itself did not significantly alter EPSP properties. No significant increases in EPSP amplitude or area were observed during the Down-state as a result of pairing EPSPs with Up-states alone (not shown). Next, we bath-applied 10 nM, 100 nM, or 10 µM DA for 5 minutes and continued to evoke Up-states via hippocampal stimulation and/or trains of local EPSPs. Typically, sweeps in which EPSP trains were paired with Up-states, and sweeps in which EPSP trains were stimulated alone were alternated until at least 10 (but typically more than 20) repetitions were obtained for each condition. At the end of the experiment, AMPA and GABA receptor antagonists were bath applied as described above to determine the nature of the local synaptic inputs.

Figure 7 summarizes the effects of DA on trains of EPSPs in the Down-state. For the group that received 100 nM DA, a two-way ANOVA with repeated measures showed a significant interaction between the pulse number in the train and the effect of DA modulation (F = 4.72; P<0.0001, df = 9). *Post-hoc* comparisons using paired t-tests showed that both the amplitude and area under the EPSP (c.f. insert in Fig. 7A) differed between the baseline and DA condition, and that these changes became significant after the 4^th^ pulse in the train. While EPSPs in the train showed synaptic depression under baseline conditions, DA application markedly increased the amplitude and area of later EPSPs in the train. These results are consistent with our own previous data from acute slices that suggested that DA could enhance the effectiveness of strong continuous inputs over single or brief stimuli [32], [42].

**Figure 8:**
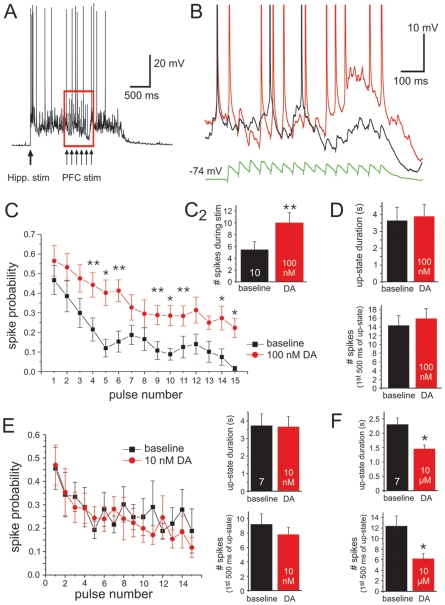
Dopamine enhances EPSP-spike coupling at moderate concentrations. A) Example trace illustrating the recording set-up used to test DA modulation of EPSPs during active network states. Up-states were evoked by stimulation of the hippocampus in PFC-Hipp-VTA co-cultures. After a minimum of 500 ms (but typically between 1000–1500 ms) into the Up-state, trains of EPSPs (15 pulses at 20 Hz, indicated by the red box) were evoked by local stimulation of afferents close to the neuron recorded in the PFC using the same neurons and stimulation parameters as shown in Figure 7 for the Down-state (N = 10). B) Representative traces showing the effectiveness of EPSPs to induce action potential firing during the Up-state under the baseline (black trace) and 100 nM DA condition (red trace). The green trace shows the averaged synaptic response during the Down-state before DA application. C) Summary graph showing the overall increase in spike number during the period of synaptic stimulation C2) and the change in EPSP-spike coupling in the 100 nM DA condition (red symbols) over baseline (black symbols). The plot shows for each pulse in the train the probability that a spike occurred within 10 ms of the onset of the stimulation. In the 100 nM DA condition the probability that an EPSP evoked an action potential was generally increased across all pulses. The relative magnitude of this effect was greater at later pulses in the train, with pairwise comparisons showing significant increases in spike probability over baseline starting at the 4^th^ pulse. D) In contrast to the effects during synaptic stimulation, bath application of 100 nM DA had no significant overall effect on Up-state duration (top) or the number of spikes before local synaptic stimulation (during the first 500 ms of the Up-state). *Post-hoc* comparisons used paired t-tests after repeated measures ANOVA (Bonferroni-adjusted level of significance * *P*<0.0033, and ** *P*<0.00165). E) The low dose of 10 nM DA had no effect of EPSP spike-coupling during the Up-state, or the overall properties of the Up-state (the inserts show measures for total Up-state duration, top bar graph; or number of spikes during the period before local synaptic stimulation, bottom; n = 7). F) In contrast, the high concentration of DA (10 µM) significantly reduced both Up-state duration (top) and the number of spikes within the Up-state. Thus, under these conditions both the synaptic signal (c.f. Fig. 7) as well as the background network activity were reduced. For comparisons at all concentrations the same cells were used as in Figure 7. In the 10 µM DA condition one cell dropped out because the Up-states evoked by hippocampal stimulation were too brief to allow stimulation of EPSP trains during the Up-state.

This result implies that in the context of an active, spiking network, DA receptor activation would be predicted to increase the effectiveness of EPSPs to evoke action potentials. To test this hypothesis directly, we examined the number of spikes in the Up-state that occurred during the train of EPSPs before and after application of 100 nM DA. We examined both the change in the total number of spikes evoked over the 750 ms of stimulation and, more specifically, the likelihood that a spike occurred within a narrow window (10 ms) following EPSP onset (EPSP-spike coupling). Consistent with results shown in Figure 4, bath application of 100 nM DA had no significant effect on Up-state properties in PFC-Hipp-VTA co-cultures. This low dose of DA affected neither the duration of Up-states nor the number of spikes that occurred within the first 500 ms of the Up-state before local synaptic stimulation occurred (Fig. 8D). In stark contrast, the number of spikes was significantly increased above baseline during the period of local synaptic stimulation in the presence of 100 nM DA. Figure 8C shows the probability that a spike occurred within a 10 ms window following local synaptic stimulation under baseline conditions (black trace) and in the presence of 100 nM DA (red trace). The insert (C2) shows the change in the absolute number of spikes during the stimulation period over baseline values, including spikes that fell outside our strict 10 ms criteria for EPSP-spike coupling. For each cell (n = 10) at least 10 repetitions (but typically more than 20) were averaged for each condition. A two-way ANOVA with repeated measures revealed 2 significant main effects: An effect of pulse number (lower spike probabilities at later pulses; F = 20.49; P<0.001), which most likely reflected the reduced synaptic strength due to synaptic depression during repetitive stimulation; and a main effect of drug application (F = 101.49; P<0.0001), which was evident as an overall increase in the probability that EPSPs were closely followed by a spike (i.e. an upward-shift in the curve). *Post-hoc* comparisons using paired t-tests showed that the relative difference in the curves became significant starting with the 4^th^ pulse (Bonferroni-adjusted level of significance of *P*<0.0033). However, as can be seen in Figure 8C, the probability that EPSPs evoked a spike varied considerably throughout the duration of the 15 pulse train.

Taken together, these observations demonstrate that in slice co-cultures, activation of DA receptors can induce a similar shift in EPSP short-term plasticity of PFC pyramidal cells as was previously observed in acute slices of the PFC. Specifically, in the Down-state, DA augmented the depolarization produced by EPSPs late in the train relative to baseline values. In the Up-state, this effect was paralleled by increased effectiveness of EPSPs to evoke spikes. The relative magnitude of this effect over baseline conditions tended to be greatest late in the stimulus train. In PFC-Hipp-VTA co-cultures, this selective enhancement of EPSP-spike coupling during patterned activity was independent of changes in the Up-state properties overall. Importantly, this increase in the effects of a synchronous signal is perfectly consistent with the previously theorized DA-mediated increase in gain.

Finally, we examined the effects of a low (10 nM) and high (10 µM) concentration of exogenous DA on locally evoked EPSPs during the Down-state and Up-state, respectively. As shown in Figure 7D-E, 10 nM DA had no effect on EPSP amplitude or area under the curve in trains of EPSPs evoked in isolation in PFC-Hipp-VTA co-cultures. Similarly, when EPSPs were evoked during Hipp-evoked Up-states, 10 nM DA did not affect the likelihood that EPSPs evoked spike firing, or the properties (duration and number of spikes) of the Up-state itself (Fig. 8E). In contrast, a high concentration of DA attenuated both the EPSPs and the Up-state. Specifically, 10 µM DA decreased the peak amplitude of the EPSP during the Down-state (F = 5.66; P<0.05; df = 7; Fig. 7F). The area under the curve was affected in a similar way, but these changes did not reach significance (Fig. 7G). More importantly, the high dose of DA reduced the duration and number of spikes in Up-states evoked by Hipp stimulation, replicating the effects seen in Up-states evoked by VTA stimulation (Fig. 8F). In the majority of cells, the duration of the Up-states were reduced to such an extent that the locally evoked train of EPSPs and the Up-state no longer completely overlapped, rendering an analysis of the effects of high DA on EPSP-spike coupling moot. Therefore, while low doses of DA had little effect, moderate concentrations of DA appeared to optimize signal gain while higher levels of DA significantly reduced gain by attenuating both the signal and the background firing. These data are therefore consistent with the proposed inverted-U profile for DA actions on PFC function [43].

## Discussion

We used organotypic slice co-cultures to show that DA modulates recurrent synaptic activity in the PFC in a concentration-dependent manner. In the presence of functional DAergic inputs from the VTA, high (≥1 µM) concentrations of exogenous DA reduced Up-states in the PFC and diminished EPSPs evoked during the Down-state, while lower doses had no effect. In contrast, in corticolimbic co-cultures lacking VTA DAergic inputs, and in VTA-PFC-Hipp cultures in which DA was depleted by reserpine/AMPT, Up-states could be enhanced by low doses of exogenous DA that had no effect in VTA containing cultures. We also demonstrate that within a narrow range of concentrations, DA selectively increased the efficiency of a train of excitatory synaptic inputs without affecting the background network activity.

As detailed below, we propose that the presence or absence of an ambient DA tone can impact a variety of physiological mechanisms that together determine a dynamic range of network responses to transient elevations of DA. However, we note that the concentrations that made up this range in our study likely reflect properties of our model system and they might be affected by differences in the pattern and density of the cortical innervation by DA fibers and alterations at the DA receptors. We used bath application of known concentrations of DA to minimize variability across cultures and to have clearly defined groups of concentrations for the comparison of DA effects. However, results from experiments in which we enhanced endogenous DAergic transmission through bath application of cocaine not only replicated the results observed with bath applied DA, but closely mimicked effects seen in adult animals in-vivo [44], [45], providing evidence that in our model system physiologically relevant levels of DA are released upon VTA stimulation. Thus, while the absolute concentrations of DA that produce effects in intact animals may differ from those used here, their relative position on the DA dose-response curve may be comparable. Another potential caveat for the interpretation of our data may be age-related changes in DA function over development as they have recently been shown for the effects of D2 receptor stimulation in interneurons [46]. Clearly, our co-culture system can not replicate developmental changes that may occur only after puberty; however, given that D2 receptors in the PFC are preferentially activated by higher DA concentrations [8], [10] an additional D2 receptor-mediated increase in interneuron firing as described by Tseng and O'Donnell [46] would likely only serve to reinforce the reduction in up-state activity described here.

Dopamine modulation of synaptic and ionic currents that govern spike initiation and repetitive firing has long been studied in isolation (for review see [8], [47]). Such studies carried out in acute slices and dissociated cells have suggested that the effect of DA receptor activation on evoked firing is membrane state-dependent [47]–[49]. Here, we replicated a main finding of these studies showing that DA increases action potential firing evoked by somatic current injection over a wide range of concentrations. Importantly, we further demonstrate that these effects could be dissociated from effects on Up-states, as the Up-states in these same cells were significantly shortened by high doses of DA. This highlights the fact that the effects of DA on intrinsic membrane excitability and synaptic connections between various cell-types in a recurrent network cannot be easily predicted from observations of each of these components in isolation.

The ability to generate multiple states of activity within local and long-distance recurrent networks is a basic feature of cortical networks [35], [50]. Reverberating synaptic activity and Up-states appear to be an emergent property of networks of a certain size and degree of connectivity [35], [51]. Up-states in-vivo and in-vitro are generated through local recurrent synaptic excitation that is balanced and controlled by the activity of GABAergic interneurons [12], [21], [52], [53]. This ongoing activity in the network can influence the response characteristics of individual neurons, serving an important role in the tuning of network processes [54]–[58]. In the waking state, the cerebral cortex generates self-sustained spontaneous “background” activity that is similar to and mechanistically related to a persistent Up-state [11], [59].

In-vivo, the occurrence of up-states in the PFC is synchronous with activity in the VTA [60] and stimulation of the VTA can induce up-states in the PFC, and their duration can be significantly shortened through systemic application of a D1 antagonist [61]. Furthermore, activity resembling Up-states can be evoked by co-application of a D1 agonist and NMDA to acute PFC slices [62]–[64]. These data highlight the important synergistic roles of glutamate and DA in regulating network activity in the PFC. The present results build on these findings by again showing that glutamate is responsible for the generation and maintenance of the Up-states while increases in DA levels over the background tone tend to reduce the Up-state once it is evoked.

Computationally, the variable recurrent activity of the Up-state enhances neuronal responsiveness to a wide range of inputs [12], [21], [55], [65], [66]. In our data, a train of synaptic inputs that was ineffective in evoking spikes from the Down-state did evoke spikes when delivered during an Up-state. This type of behavior is reminiscent of stochastic resonance [67]–[70] whereby noise enhances signal transmission in moderate regimes, but is detrimental if noise levels are too high or signals are too small. As a result, in order to effectively control the gain within active cortical networks, it would be beneficial to modulate Up-states (background) and synchronous inputs (signals) independently. Catecholamines, and particularly DA, are believed to increase the efficiency of cortical processing by augmenting the signal to noise (S∶N) ratio, or the gain within cortical networks [4], [37]–[39]. Specifically, it has been hypothesized that by increasing the effects of strong, sustained depolarizing inputs relative to background firing [40], [41], [71], DA augments task-related activity (i.e. a “signal”) in working memory [1], [3]–[5]. At higher DA levels, S∶N degrades due to an overall suppressive effect on both S and N [5]. The present data show that DA can modulate both a signal and network background activity (the Up-state) in a concentration-dependent manner. Importantly, there existed an intermediate range of DA concentrations at which a synaptic signal was selectively enhanced without affecting global network activity.

Very high or very low levels of DA (i.e. supranormal concentrations of DA higher than those that could be evoked by synaptic stimulation of the VTA, or the lack of a tonic DA level, respectively) both affected the global network activity, either by directly modulating Up-states or by influencing how Up-states responded to transient applications of DA. High concentrations of DA consistently reduced network activity during the Up-state, regardless of whether the cultures possessed an intrinsic source of DA innervation from the VTA or not (Figs. 3 and 6). This effect mirrored the suppressive action of high levels of DA on trains of synaptic inputs evoked during the Down-state (Fig. 7). The detrimental effects of supranormal concentrations of DA on VTA-evoked Up-states were replicated by bath application of cocaine that increases extracellular DA content by blocking DA reuptake and enhancing DA release [72]. These convergent lines of evidence suggest that when a certain cumulative level of DA is exceeded, a reduction in network activity occurs. In contrast, when no tonic release of DA was present the dose-response curve was altered such that in corticolimbic cultures that lacked VTA DA neurons Up-states were enhanced by moderate DA concentrations, which had no effect when delivered to VTA-containing cultures. The background tone therefore appears to alter the response to subsequent more phasic release of DA as originally suggested by Grace [6]. Functionally, the low nM background DA may act to constrain changes in general excitability and prevent subsequent transient or phasic elevations of DA from enhancing network “noise”.

The present results support the idea that DA modulation of active networks follows an inverted-U dose-response curve [43], [73]. In the original formulation of that theory, persistent activity related to working memory was said to be optimized by moderate D1 receptor activation while either very weak or supranormal stimulation of D1 receptors had detrimental effects [43], [73]. The present results expand on this idea in the following ways: Low background extrasynaptic DA levels appear to prevent the increases in excitability that would otherwise occur in response to a phasic elevation in DA (Fig. 6). Moderate elevations of DA above the tonic background levels appear to bring the system to the peak of the inverted U-curve where signals are potently increased without affecting noise, thereby optimizing S∶N (Fig. 8). In our data, an intermediate concentration of DA (100 nM) increased both the depolarization produced by the train of inputs, as well as EPSP-spike coupling during the Up-state In acute PFC brain slices, DA similarly modulated short-term synaptic plasticity of EPSPs onto primate interneurons [42] and rodent pyramidal cells [32]. In pyramidal cells (but not in interneurons) this effect depended on NMDA receptor activation and was hypothesized to promote persistent firing [32]. Finally, the high levels of DA on the far right hand side of the hypothesized inverted U curve decrease both signal and noise, effectively quelling overall PFC activity, which is similar to what is observed in the behaving animal [5]. This type of differential modulation of signal and noise at varying DA levels is predicted by computational models that simulate the known effects of DA on AMPA, GABA and NMDA currents [40], [41], [74]–[76].

The results of the present study show in a biological system that the hypothesized concentration-dependent effects of DA combine in a manner that is consistent with an inverted U-curve of DA function and directly demonstrate a role of DA in S∶N modulation as predicted by theoretical models. The combination of theoretical and experimental approaches may allow us to better define this curve and provide new insights into the normal function of the mesofrontal DA system as well as its possible dysfunction in the pathophysiology of schizophrenia, chronic stress, or drug addiction.
